# Transcolonic endoscopic appendectomy via snare-through-line resection for stump appendicitis

**DOI:** 10.1055/a-2357-2483

**Published:** 2024-07-26

**Authors:** Yun Wang, Ming-Yan Cai, Sheng-Li Lin, Mo-Fan Li, Ping-Hong Zhou

**Affiliations:** 1 92323 Endoscopy Center and Endoscopy Research Institute, Zhongshan Hospital Fudan University, Shanghai, China; 2 Shanghai Collaborative Innovation Center of Endoscopy, Shanghai, China; 3 92323 Nursing Department, Zhongshan Hospital Fudan University, Shanghai, China

A 16-year-old girl was admitted due to recurrent abdominal pain 10 months after laparoscopic appendectomy. Physical examination showed tenderness at the McBurney point. Computed tomography revealed a distended appendiceal stump with peristump inflammation and dense suture material at the ligated end. After comprehensive preoperative assessment, transcolonic endoscopic appendectomy was chosen.

Following submucosal injection, a hook knife was employed to create a mucosal incision and perform partial dissection of the submucosal layer, succeeded by full-thickness resection with an IT knife ( Fig. 1 , Video 1 ). The endoscope was advanced into the peritoneal cavity, revealing a dilated appendiceal remnant with fibrous adhesions. The adhesions encircling the appendiceal stump were loosened by blunt dissection or with the IT knife. A clip with a 6–0 Prolene suture attached was mounted on the endoscope. The clip was then advanced with the endoscope and deployed at the end of the appendiceal stump. Next, a snare was inserted through the line and the working channel of the endoscope. Using the snare-through-line method, the appendiceal stump was completely resected. Mesenteric vessels were coagulated with hot biopsy forceps. The cecal defect was closed with a novel detachable over-the-scope clip (Senscure, China). The twin grasper facilitated precise alignment of the defect edges. A decompression tube was secured in the cecum with dental floss and an endoclip. The total procedure duration was 70 minutes. The patient had an uneventful recovery and was discharged 4 days after the procedure. Histopathology confirmed acute and chronic stump appendicitis.

**Fig. 1:**
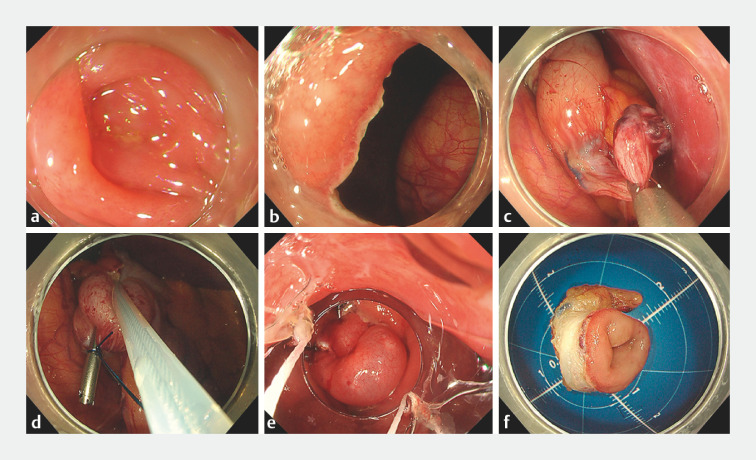
Endoscopic images of transcolonic endoscopic appendectomy for stump appendicitis. **a** Appendiceal orifice. **b** Full-thickness resection around the appendiceal orifice. **C** A dilated appendiceal stump with suture line from the first appendectomy. **d** Snare-through-line resection of the appendiceal stump. **e** Defect closure with a novel detachable over-the-scope clip. **f** The resected appendiceal stump.

Transcolonic endoscopic appendectomy via snare-through-line resection for stump appendicitis.Video 1

Stump appendicitis is a rare late complication after appendectomy, with an incidence presumed to be between 0.002% and 0.15% 1 2 . Using the snare-through-line method, we successfully performed endoscopic resection for stump appendicitis, expanding the horizon of natural orifice transluminal endoscopic surgery. Further clinical studies and long-term follow-ups are warranted to substantiate the safety and efficacy of this procedure.

Endoscopy_UCTN_Code_TTT_1AQ_2AD_3AF

## Notes

Endoscopy E-Videos https://eref.thieme.de/e-videos
*E-Videos* is an open access online section of the journal *Endoscopy* , reporting on interesting cases and new techniques in gastroenterological endoscopy. All papers include a high-quality video and are published with a Creative Commons CC-BY license. Endoscopy E-Videos qualify for HINARI discounts and waivers and eligibility is automatically checked during the submission process. We grant 100% waivers to articles whose corresponding authors are based in Group A countries and 50% waivers to those who are based in Group B countries as classified by Research4Life (see: https://www.research4life.org/access/eligibility/ ). This section has its own submission website at https://mc.manuscriptcentral.com/e-videos .
